# The effect of succinylcholine on malignant hyperthermia events in susceptible swine

**DOI:** 10.1186/1471-2253-14-14

**Published:** 2014-03-07

**Authors:** Frank Schuster, Stephan Johannsen, Susanne Moegele, Thomas Metterlein, Norbert Roewer, Martin Anetseder

**Affiliations:** 1 Department of Anaesthesia and Critical Care, University of Wuerzburg, Oberduerrbacher Straße 6, D-97080 Wuerzburg, Germany; 2 Department of Anaesthesiology, University of Regensburg, Regensburg, Germany; 3 Department of Anaesthesia, Hospital Landshut-Achdorf, Landshut, Germany

**Keywords:** Malignant hyperthermia, Succinylcholine, Halothane, Swine

## Abstract

**Background:**

While the impact of volatile anaesthetics to induce malignant hyperthermia (MH) is abundantly clear, the role of succinylcholine still remains controversial. To evaluate the influence of succinylcholine on porcine MH events, the authors investigated the hemodynamic and metabolic responses in MH susceptible (MHS) and non-susceptible (MHN) swine following either succinylcholine or halothane application alone or a combination of both substances.

**Methods:**

With approval of the local animal care committee 27 MHS and 30 MHN pigs were anaesthetized and mechanically ventilated. Fiberoptic probes for continuous PCO_2_ measurement were inserted into the femoral vein and the triceps muscle. Group A received succinylcholine 4 mg/kg, group B incremental doses of halothane (0.5, 1.0 vol%) and group C succinylcholine and halothane simultaneously. Vital signs were recorded continuously.

**Results:**

Prior to drug application measured values did not differ between MHS and MHN. While MHN pigs did not show relevant alterations, succinylcholine, halothane and the combination of both lead to significant hemodynamic and metabolic changes in MHS swine.

**Conclusions:**

Hemodynamic and metabolic alterations following succinylcholine were similar to halothane in MHS pigs. The combination of both pharmacological agents potentiated the observed effects. According to these results succinylcholine acted as an independent and supportive factor during onset of an MH episode.

## Background

Malignant hyperthermia (MH) is a potentially lethal pharmacogenetic disorder characterized by a disturbance of skeletal muscle calcium homeostasis. In predisposed humans and animals exposure to triggering agents may lead to a hypermetabolic muscular syndrome. Uncontrolled sarcoplasmic calcium release caused by mutations in the ryanodine receptor subtype 1 or in the dihydrophyridine receptor results in hypoxemia, hypercapnia, tachycardia, muscular rigidity, acidosis, hyperkalaemia and hyperthermia [1]. While the impact of volatile anaesthetics in the development of MH is abundantly clear, the role of the depolarising muscle relaxant succinylcholine (SCh) still remains controversial [2]. For instance, application of commonly used halogenated anaesthetics induces pathological muscular contractions in muscle bundles of susceptible patients *in vitro*[3], whereas SCh did not induce a reproducible muscular response [4]. Furthermore, reliable reports of fulminant MH episodes in humans or animals induced by administration of SCh alone are lacking in the current literature [2].

Hence, the aim of the present study was to investigate the hemodynamic and metabolic response in MH susceptible (MHS) and non-susceptible (MHN) pigs following application of SCh, halothane or the combination of both pharmacological agents, to evaluate the influence of SCh on porcine MH events.

## Methods

### Experimental protocol

With approval of the local animal care committee (Government of Unterfranken, Wuerzburg, Germany, application number: 44/99; 14/05), 57 pietrain pigs weighing 30.6 (27.8 – 31.5) kg were examined. Prior to the investigation, MH susceptibility or wild type was determined by DNA analysis regarding the presence of a homozygous arginine 615 mutation of the ryanodine receptor indicating MHS.

Anaesthesia was induced intravenously via an ear vein with thiopental (14 – 17 mg/kg) and maintained using a midazolam (0.2 - 0.4 mg/kg/h) and fentanyl (0.01 - 0.04 mg/kg/h) infusion in all swine. After the trachea was intubated (7.0 mm ID endotracheal tube, Rüsch, Kernen i.R., Germany) without the use of muscle relaxants, ventilator settings (Servo 900C, Siemens, Erlangen, Germany) were adjusted to keep an end-tidal carbon dioxide partial pressure (PCO_2_) of 30–35 mmHg (respiratory rate: 12 – 14/min; tidal volume: 10 – 15 mL/kg; positive end expiratory pressure: 5 mmHg; 50% oxygen and 50% air). Afterwards, two PCO_2_ probes (ParaTrend 7+, Diametrics Medical Inc., High Wycombe, Buckinghamshire, UK) were placed under ultrasound guidance into the left triceps muscle and into the left femoral vein of the animals. Vital signs of the animals were monitored continuously by mean arterial blood pressure (MAP) in the saphenous artery, peripheral oxygen saturation (SaO_2_), ECG and rectal temperature. A warming blanket and an infrared lamp were used to prevent the animals from hypothermia. In 15 minutes intervals arterial blood samples were obtained and immediately analysed for pH, arterial oxygen partial pressure (PaO_2_), carbon dioxide partial pressure (PaCO_2_), base excess (BE) and lactate. Throughout the experiment 4 mL/kg/h Ringer solution (B. Braun, Melsungen, Germany) was administered via a central venous line placed in the right jugular vein.

After stable conditions were achieved, animals were subdivided into three groups. Following baseline recording, the SCh group (MHS: n = 9; MHN: n = 10) received two single boluses of SCh 4 mg/kg (Nycomed Pharma GmbH, Unterschleissheim, Germany) at intervals of 15 minutes, while in the halothane group (MHS: n = 6; MHN: n = 10) the anaesthetic vapor setting was adjusted to achieve an end-tidal halothane concentration of 0.5 vol% for the first 15 minutes, followed by an elevation to 1 vol% for further 15 minutes. In a third group (MHS: n = 12; MHN: n = 10) a single bolus of SCh 4 mg/kg combined with an end-tidal halothane concentration of 0.5 vol% was applied at first and was augmented after 15 minutes by another SCh bolus 4 mg/kg and an increase of halothane to 1 vol%. Different sizes of the investigated groups were caused by the availability of the animals. Intramuscular and femoral venous PCO_2_ were recorded in 1-minute intervals. Arterial blood samples were analysed before and after drug application. Systemic hemodynamic parameters were monitored throughout the experiment. The clinical occurrence of MH was defined as the development of at least four out of six conditions: arterial pH ≤ 7.20, PaCO_2_ ≥ 50 mmHg, arterial BE ≤ −5 mmol/l, lactate ≥ 5 mM, end-tidal PCO_2_ ≥ 45 mmHg and an increase of rectal core temperature ≥ 38.5°C [5].

### Carbon dioxide measurements

The fiberoptic PCO_2_ sensor consisted of an optode, kept in a heparin-coated microporous polyethylene tube of approximately 0.5 mm in diameter and permeable to the substance to be measured. The cylindrical sensor construction allowed measurements over the entire probe surface. Prior to insertion in the target tissue, the probes were calibrated *in vitro* by exposure to different standard gas concentrations (TrendCare Calibrator, Diametrics Medical Inc., High Wycombe, Buckinghamshire, UK).

### Statistical analysis

Data are displayed as median and interquartile range. Statistical comparisons between and within the investigated groups were performed by repeated measures ANOVA and post-hoc Tukey test. A value of p < 0.05 was considered statistically significant.

## Results

### Clinical occurrence of MH

According to the predefined metabolic parameters none of the MHN swine developed any clinical signs of an MH episode after application of SCh, halothane or both agents. In contrast, MH occurred in all MHS animals of the halothane and the SCh plus halothane group, while only four out of nine MHS pigs in the SCh group fulfilled the defined criteria.

### Hemodynamic data

Prior to drug application hemodynamic variables and rectal temperature were comparable between the investigated groups. While in MHN swine SCh had little or no effect on hemodynamic parameters, relevant hypotension, tachycardia and an increase in end-tidal PCO_2_ were observed in all MHS animals. However, SCh did not induce a rise of body temperature in the MHS pigs.

Interestingly, incremental concentrations of halothane caused a significant decrease of arterial blood pressure in both investigated groups, while increases of end-tidal PCO_2_ only became significant in MHS swine. But, neither heart rate nor rectal temperatures were significantly affected by halothane application in normal or susceptible animals.

In contrast, the effects of halothane combined with SCh were numerous and pronounced. Beside distinctive hypotension and tachycardia, a significant temperature increase was noticed in MHS pigs. Furthermore, changes in end-tidal PCO_2_ were significantly different between susceptible and normal animals. It is noteworthy, that eleven of the twelve MHS pigs immediately died after reinjection of SCh and raise of end-tidal halothane concentration to 1 vol%. Even MHN pigs developed significant hypotension and tachycardia after administration of halothane plus SCh (Table 1).

**Table 1:** Hemodynamic variables of malignant hyperthermia susceptible (MHS) and non-susceptible (MHN) pigs following succinylcholine or halothane or succinylcholine and halothane application

**Diagnose**	**Time [min|**	**HR [bpm]**	**MAP [mmHg]**	**SaO**_**2 **_**[%]**	**etCO**_**2 **_**[mmHg]**	**Temperature [°C]**
MHS_SCh_ (n = 9)	0 min	53 [52–61]	84 [83–89]	98 [98–98]	35 [31–35]	37,3 [37,1-37,3]
	15 min	58 [53–65]	85 [82–89]	98 [97–98]	35 [32–36]	37,2 [37,0-37,3]
	30 min	73 [63–85]*	67 [55–74]*	98 [97–98]	48 [42–51]*^#^	37,5 [37,2-37,5]
	45 min	70 [68–92]*	66 [60–76]*	98 [97–98]	54 [44–62]*^#^	37,6 [37,3-37,9]
MHN_SCh_ (n = 10)	0 min	67 [63–72]	75 [70–86]	99 [97–100]	33 [30–35]	37,0 [36,8-37,5]
	15 min	71 [63–75]	74 [67–81]	99 [96–100]	33 [30–35]	36,9 [36,7-37,4]
	30 min	72 [67–75]	72 [68–76]	99 [98–100]	34 [31–34]	37,0 [36,6-37,4]
	45 min	66 [61–70]	71 [69–76]	99 [97–100]	34 [31–35]	37,0 [36,6-37,4]
MHS_Halothane_ (n = 6)	0 min	61 [60–66]	82 [75–84]	98 [98–99]	33 [30–35]	37,4 [36,9-37,5]
	15 min	58 [55–66]	82 [76–87]	98 [97–99]	33 [30–34]	37,4 [36,9-37,4]
	30 min	61 [59–64]	49 [45–53]*^#^	98 [97–99]	36 [32–40]*	37,3 [37,0-37,5]
	45 min	76 [61–110]	41 [38–45]*	97 [95–98]	55 [44–65]*	37,5 [37,1-37,9]
MHN_Halothane_ (n = 10)	0 min	64 [57–70]	72 [70–79]	98 [98–100]	33 [31–35]	36,8 [36,6-37,3]
	15 min	64 [55–69]	73 [68–80]	98 [97–99]	35 [31–35]	37,0 [36,8-37,2]
	30 min	68 [61–76]	66 [55–72]*	99 [98–100]	33 [31–34]	37,1 [36,6-37,2]
	45 min	69 [62–73]	51 [45–56]*	99 [97–100]	31 [29–33]*^#^	37,0 [36,8-37,3]
MHS_Halothane+SCh_ (n = 12)	0 min	65 [55–87]	60 [59–72]	98 [98–99]	31 [30–32]	37,5 [37,4-37,9]
	15 min	80 [70–92]*	60 [55–75]	98 [97–99]	33 [32–34]*	37,5 [37,3-37,8]
	30 min	168 [113–204]*^#^	41 [35–45]*	96 [95–98]*	63 [55–65]*^#^	38,0 [37,6-38,2]*^#^
	45 min	200+	60+	98+	79+	39,5+
MHN_Halothane+SCh_ (n = 10)	0 min	71 [61–79]	67 [60–80]	99 [97–99]	33 [31–33]	37,6 [37,1-37,8]
	15 min	83 [73–97]*	61 [60–78]	98 [97–99]	32 [31–34]	37,2 [37,1-37,6]*
	30 min	93 [84–102]*	57 [53–60]*	98 [96–98]	31 [30–32]	37,0 [36,8-37,5]*
	45 min	100 [95–105]*	52 [48–60]*	96 [95–100]	32 [30–32]	36,9 [36,7-37,4]*

t = 0 minutes: Baseline values; t = 15 minutes: Application of 4 mg/kg succinylcholine or 0.5 vol% halothane or both; t = 30 minutes: Application of 4 mg/kg succinylcholine or 1 vol% halothane or both.

HR = heart rate; MAP = mean arterial blood pressure; SaO_2_ = peripheral oxygen saturation; etCO_2_ = end-tidal carbon dioxide; SCh = succinylcholine.

Data as median and interquartile range;

*significant changes from baseline values; ^#^significant differences between MHS and MHN; p < 0.05. +: data from only one MHS animal.

### Metabolic data

#### Arterial blood gas samples

There were no significant metabolic differences in arterial blood samples prior to SCh, halothane or SCh and halothane application between MHS and MHN swine. With exception of minor PaO_2_ alterations, neither SCh nor halothane or the combination of both agents resulted in serious changes of metabolic variables in the MHN groups. However, in MHS animals SCh alone caused a significant and severe decrease in pH and BE and a relevant increase of PaCO_2_ and serum lactate. Systemic halothane aggravated the metabolic alterations in the MHS group. Furthermore, simultaneous application of halothane and SCh resulted in an earlier and more distinctive metabolic deterioration and was lethal for the majority of the MHS pigs (Table 2).

**Table 2:** Arterial blood gas samples of malignant hyperthermia susceptible (MHS) and non-susceptible (MHN) pigs following succinylcholine or halothane or succinylcholine and halothane application

**Diagnose**	**Time [min|**	**pH**	**PaO**_**2 **_**[mmHg]**	**PaCO**_**2**_**[mmHg]**	**BE [mmol/l]**	**Lactate [mM]**
MHS_SCh_ (n = 9)	0 min	7,38 [7,36-7,44]	260 [242–285]	40 [38–43]	0,5 [−1,7-1,2]	0,5 [0,4-0,7]
	15 min	7,39 [7,37-7,44]	282 [263–298]#	40 [38–42]	0,0 [−1,7-1,2]	0,5 [0,4-0,6]
	30 min	7,21 [7,11-7,22]*^#^	243 [194–269]	59 [55–66]*^#^	−4,6 [−9,2- -3,6]*^#^	2,7 [2,2,3,2]*^#^
	45 min	7,19 [7,11-7,23]*^#^	240 [229–255]	64 [57–72]*^#^	−3,7 [−9,2- -3,6]*^#^	1,5 [1,2-1,8]*^#^
MHN_SCh_ (n = 10)	0 min	7,42 [7,37-7,45]	246 [187–274]	42 [39–43]	2,0 [−0,2-2,6]	0,7 [0,5-0,9]
	15 min	7,42 [7,37-7,43]	249 [197–266]	42 [40–45]	1,8 [−0,4-2,5]	0,6 [0,5-0,7]
	30 min	7,40 [7,36-7,44]	233 [183–271]	43 [39–46]	2,7 [0,3-3,0]	0,6 [0,5-1,0]
	45 min	7,39 [7,36-7,44]	253 [198–268]	44 [39–46]	1,4 [−0,4-2,4]	0,6 [0,5-0,7]
MHS_Halothane_ (n = 6)	0 min	7,38 [7,34-7,39]	260 [257–270]	40 [39–41]	−1,2 [−2,5- -0,4]	0,6 [0,5-0,7]
	15 min	7,39 [7,36-7,42]	265 [257–265]	39 [38–40]	−0,2 [−1,5-0,4]	0,5 [0,4-0,6]
	30 min	7,29 [7,19-7,38]	254 [248–259]	48 [41–52]	−3,2 [−8,9- -0,4]	2,4 [1,7-5,1]
	45 min	7,03 [6,99-7,09]*^#^	212 [192–247]	67 [58–77]*^#^	−12,3 [−15,1- -10,8]*^#^	9,0 [8,2-9,9]*^#^
MHN_Halothane_ (n = 10)	0 min	7,39 [7,35-7,42]	247 [201–279]	43 [39–46]	0,5 [−1,5-1,1]	0,6 [0,5-0,8]
	15 min	7,38 [7,33-7,42]	249 [195–276]	43 [39-46|	0,0 [−1,8-0,9]	0,8 [0,6-1,0]
	30 min	7,39 [7,35-7,49]	238 [200–253]	42 [38–46]	−0,3 [−2,2-0,8]	0,9 [0,5-1,1]
	45 min	7,39 [7,34-7,40]	232 [179–259]*	40 [39–44]	−1,0 [−2,6-0,6]	0,6 [0,5-1,0]
MHS_Halothane+SCh_ (n = 12)	0 min	7,47 [7,41-7,51]	256 [250–281]	36 [32–37]	2,4 [0,3-4,6]	1,1 [0,9-1,4]
	15 min	7,42 [7,33-7,44]*	261 [247–271]	39 [38–44]*	0,1 [−2,6-2,1]	3,1 [1,6-3,6]
	30 min	6,90 [6,83-7,13]*^#^	256 [194–314]^#^	69 [33–87]*^#^	−18,6 [−23,3- -15,0]*^#^	15,7 [15,3-21,4]*^#^
	45 min	6,69+	247+	42+	−34,1+	29,5+
MHN_Halothane+SCh_ (n = 10)	0 min	7,41 [7,41-7,48]	262 [242–291]	36 [35–38]	1,0 [−1,9-3,3]	1,1 [0,9-1,4]
	15 min	7,44 [7,37-7,46]	243 [231–274]	37 [36–41]	0,3 [−21-2,8]	1,1 [0,9-1,4]
	30 min	7,38 [7,35-7,44]	238 [216–274]	37 [36–38]	−0,4 [−3,6-1,5]	1,3 [1,0-1,4]
	45 min	7,37 [7,34-7,43]	239 [205–253]*	39 [37–40]	−2,3 [−4,7- -0,5]	1,2 [1,1-1,3]

t = 0 minutes: Baseline values; t = 15 minutes: Application of 4 mg/kg succinylcholine or 0.5 Vol% halothane or both; t = 30 minutes: Application of 4 mg/kg succinylcholine or 1 Vol% halothane or both.

SCh = succinylcholine; PaO_2_ = arterial oxygen partial pressure; PaCO_2_ = arterial carbon dioxide partial pressure; BE = base access.

Data as median and interquartile range.

*significant changes from baseline values; ^#^significant differences between MHS and MHN; p < 0.05. +: data from only one MHS animal.

### Muscular and femoral venous carbon dioxide measurements

Intramuscular and femoral venous baseline PCO_2_ levels before trigger application were comparable between the investigated MHS and MHN animals. In MHN pigs no differences in muscular or venous PCO_2_ values were recorded at any experimental time point following SCh and/or halothane application (Table 3 and Table 4). In contrast, intravenous SCh and systemic halothane respectively resulted in a significant increase of femoral venous PCO_2_ in MHS pigs. In addition, simultaneous application of SCh and halothane induced an even more rapid and excessive rise of venous PCO_2_ levels (Table 3). The changes of muscular PCO_2_ values were similar to the femoral venous results. SCh induced a significant, but compared to halothane a minor increase of muscular PCO_2_. Simultaneous exposure of MHS pigs to SCh and halothane aggravated the changes of muscular metabolism (Table 4).

**Table 3:** **Femoral venous carbon dioxide partial pressure (PCO**_**2**_**) of malignant hyperthermia susceptible (MHS) and non-susceptible (MHN) pigs following succinylcholine or halothane or succinylcholine and halothane application**

**Diagnose**	**Femoral venous PCO**_**2 **_**[mmHg] t = 0 min**	**t = 15 min**	**t = 30 min**	**t = 45 min**
MHS_SCh_ (n = 9)	51 [45–54]	52 49–56]	78 [67–93]*^#^	79 [72–105]*^#^
MHN_SCh_ (n = 10)	50 [43–55]	49 [44–54]	51 [45–56]	49 [43–56]
MHS_Halothane_ (n = 6)	59 [46–67]	55 [45–60]	71 [58–84]*	138 [98–152]*^#^
MHN_Halothane_ (n = 10)	50 [44–58]	52 [44–57]	54 [44–58]	54 [44–60]
MHS_Halothane+SCh_ (n = 12)	51 [47–56]	57 [53–64]	151 [110–192]*^#^	199+
MHN_Halothane+SCh_ (n = 10)	51 [44–53]	49 [44–52]	50 [47–57]	51 [47–61]

t = 0 minutes: Baseline values; t = 15 minutes: Application of 4 mg/kg succinylcholine or 0.5 vol% halothane or both; t = 30 minutes: Application of 4 mg/kg succinylcholine or 1 vol% halothane or both.

SCh = succinylcholine.

Data as median and interquartile range.

*significant changes from baseline values; ^#^significant differences between MHS and MHN; p < 0.05. +: data from only one MHS animal.

**Table 4:** **Muscular carbon dioxide partial pressure (PCO**_**2**_**) of malignant hyperthermia susceptible (MHS) and non-susceptible (MHN) pigs following succinylcholine or halothane or succinylcholine and halothane application**

**Diagnose**	**Muscular PCO**_**2 **_**[mmHg] t = 0 min**	**t = 15 min**	**t = 30 min**	**t = 45 min**
MHS_SCh_ (n = 9)	60 [45–64]	59 [45–62]	73 [65–87]*^#^	78 [69–92]*^#^
MHN_SCh_ (n = 10)	54 [41–59]	55 [46–60]	56 [47–59]	58 [50–60]
MHS_Halothane_ (n = 6)	54 [51–58]	54 [50–57]	59 [57–71]	106 [101–120]*^#^
MHN_Halothane_ (n = 10)	59 [48–63]	58 [49–62]	54 [48–63]	56 [48–60]
MHS_Halothane+SCh_ (n = 12)	56 [48–60]	56 [47–66]	114 [56–167]	140+
MHN_Halothane+SCh_ (n = 10)	56 [54–65]	56 [47–66]	56 [55–65]	58 [56–68]

t = 0 minutes: Baseline values; t = 15 minutes: Application of 4 mg/kg succinylcholine or 0.5 vol% halothane or both; t = 30 minutes: Application of 4 mg/kg succinylcholine or 1 vol% halothane or both.

SCh = succinylcholine.

Data as median and interquartile range;

*significant changes from baseline values; ^#^significant differences between MHS and MHN; p < 0.05. +: data from only one MHS animal.

Noteworthy, in MHS animals application of halothane and/or SCh resulted in a more distinctive and rapid increase of PCO_2_ in the triceps muscle and in the femoral venous blood compared to PaCO_2_ and end-tidal PCO_2_ values. (Table 2, Table 3 and Table 4).

## Discussion

The disturbance of intracellular calcium homeostasis due to an uncontrolled sarcoplasmic calcium release via mutated calcium releasing channels is widely accepted as the underlying pathophysiological mechanism of MH. The excessive increase of skeletal muscular intracellular calcium levels in MHS individuals leads to muscle rigidity, enhanced mitochondrial energy turnover with excessive oxygen consumption, carbon dioxide increase, heat production and metabolic acidosis [1,6].

Undoubtedly, all volatile anaesthetics have the ability to provoke an MH episode [2]. For instance, it is well known that halothane induces an excessive sarcoplasmic calcium release in predisposed individuals via interaction with altered ryanodine receptors subtype 1, leading to the clinical syndrome of MH [7,8]. The hemodynamic and above all the metabolic changes observed in this investigation with decrease of pH and BE combined with significant increases of end-tidal PCO_2_, PaCO_2_ and lactate following halothane exposition in MHS swine documented once again the MH inducing potency of volatiles.

In contrast, the underlying mode of action or the impact of SCh to elicit an MH remained unclear so far. Larach and colleagues rated 284 cases that were suspicious of MH and had been reported to the North American MH Registry using a clinical grading scale to evaluate the likelihood of truly being MH. Only two cases (0.7%) were regarded as ”very likely“ or ”almost certain“ being MH, after SCh had been applied to the patients but not a volatile anaesthetic [9]. Recently, a European multicentre study analysed the cases of 200 patients with history of a clinical MH episode and confirmed MH disposition by *in vitro* contracture test. In this study only two patients (1%; 1 MHS and 1 MH equivocal) with SCh induced MH crisis in absence of any inhalation anaesthetic were identified [10].

In animals, intramuscular injection of halothane resulted in a local hypermetabolic response with locally limited rise of lactate and PCO_2_, enabling a differentiation between MH susceptible and normal pigs [11], while local SCh did not initiate a metabolic reaction [12]. Furthermore, variable responses to intravenous SCh application were reported in susceptible swine. While in previous studies an MH episode could not be elicited with SCh alone [13,14], Iaizzo and colleagues demonstrated that SCh induced porcine MH [15,16]. However, the fact that acidosis and hypercapnia occurred shortly after SCh-induced hypotension and tachycardia, which were treated with intravenous saline infusion does not exclude reperfusion of temporary hypoperfused tissue to be causal for the metabolic alterations [15]. In contrast to this previous study, in the presented investigation the decrease of arterial blood pressure induced by SCh was not treated. Hence, the observed metabolic reactions, including a decrease of pH and BE and an increase of end-tidal PCO_2_, PaCO_2_ and lactate as well as muscular and femoral PCO_2_ in susceptible pigs, are likely to be signs of a developing MH. Even if the alterations after SCh were less distinctive compared to the halothane-induced effects, our results documented a severe metabolic stimulation in the susceptible animals.

Possible explanations for SCh-induced MH may be based on the pharmacological characteristics of this agent. After intravenous injection, SCh activates the nicotinergic acetylcholine receptor, which acts as an unspecific cation channel provoking a local depolarization of the cell membrane. The transient depolarization of voltage-gated receptors and an influx of extracellular calcium via the nicotinergic acetylcholine receptors followed by an increase of intracellular calcium levels may trigger MH in predisposed individuals [17]. In this context, muscular fasciculation and rigidity caused by SCh were considered to be causal for MH, since pre-treatment with non-depolarizing muscle relaxants diminished or completely prevented the appearance of an MH episode [15,16,18]. Thereby, the rise of intracellular calcium during the depolarisation period itself might be sufficient to initiate sarcoplasmic calcium release and after exceeding individual thresholds, MH occurs in affected individuals. Similar to previous studies prolonged fasciculation was seen in the investigated MHS pigs. However, the importance and the exact mode of action of SCh in setting off an MH episode are still indistinct and require further research.

In the current study, simultaneous application of halothane and succinylcholine potentiated the induced hemodynamic and metabolic effects compared to the changes following administration of halothane or SCh alone in MHS swine. Interestingly, a significant rise of body temperature only occurred after administration of a combination of both pharmacological agents, reflecting the severe metabolic breakdown. Our findings suggest an additive effect of SCh as postulated by previous investigations in which the onset of an MH episode was significantly enhanced and the increase of serum creatine kinase was more pronounced after a combination of SCh and volatile anaesthetics [10,19,20].

Hypermetabolism during a fulminant MH episode is typically associated with an excessive increase of end-tidal and arterial carbon dioxide concentrations [1,6]. The more distinctive rise of femoral venous and muscular PCO_2_ levels compared to PaCO_2_ and end-tidal PCO_2_ in MHS swine might be caused by a significant muscular venous outflow of carbon dioxide. Hence, blood gas analysis of venous blood might be as helpful as an arterial blood gas in clinical practice for real time detection of a developing MH episode.

## Conclusions

In conclusion, the findings of our investigation clearly demonstrated, that both SCh and halothane alone had the ability to induce an MH in susceptible swine. Furthermore, the observed hemodynamic and metabolic effects were excessively potentiated by combination of both pharmacological agents. Based on these results, SCh acts independent as well as supportive to provoke an MH episode in predisposed individuals. Hence, even if convincing reports of fulminant MH in humans induced by SCh alone are lacking, the obtained findings of our study proved the MH eliciting potency of SCh. However, the underlying mode of action of SCh to cause an MH episode still remains unclear and needs to be evaluated in further investigations.

## Abbreviations

BE: Base excess; IVCT: In vitro contracture test; MAP: Mean arterial blood pressure; MH: Malignant hyperthermia; MHN: Malignant hyperthermia non-susceptible; MHS: Malignant hyperthermia susceptible; PaCO_2_: Arterial carbon dioxide pressure; PaO_2_: Arterial oxygen partial pressure; PCO_2_: End-tidal carbon dioxide partial pressure; SaO_2_: Peripheral oxygen saturation; SCh: Succinylcholine.

## Competing interests

All authors declare that they have no competing interests.

## Authors’ contributions

FS conceived the study, accompanied the data acquisition, collected the data and drafted the manuscript. SJ analyzed the data and helped writing the manuscript. SM analyzed the data. TM collected the data. NR participated in the design of the study. MA participated in the design of the study, collected and analyzed the data. All authors read and approved the final manuscript.

## Pre-publication history

The pre-publication history for this paper can be accessed here:

http://www.biomedcentral.com/1471-2253/14/14/prepub
